# Stage 4S Bilateral Adrenal Neuroblastoma in a Newborn

**Published:** 2014-04-01

**Authors:** Rahul Gupta, Tariq Ahmed Mala, Praveen Mathur, Rozy Paul, Shahid Amin Mala

**Affiliations:** Department of Paediatric Surgery, SPMCHI, SMS Hospital, Jaipur Rajasthan India; Department of Surgery, ASCOMS and Hospital Jammu (J and K) India-180017; Department of Paediatric Surgery, SPMCHI, SMS Hospital, Jaipur Rajasthan India; Medical officer, Jaipur Rajasthan India; S.S. Medical College Rewa (M.P) India

**Keywords:** Stage 4S, Bilateral adrenal neuroblastoma, Neonate

## Abstract

Stage 4S bilateral adrenal neuroblastoma presenting in the neonatal period is extremely rare. A 1-day-old male with 4Sbilateral adrenal neuroblastoma complicated by marked hepatomegaly managed by chemotherapy is being reported. The provisional diagnosis of neuroblastoma was made in the fetal life during the last trimester of pregnancy. Cardiomyopathy due to doxorubicin cytotoxicity developed over ensuing years, which is being treated.

## INTRODUCTION

Stage 4S neuroblastoma (NB) represents approximately 7–10% of all NB cases.[1] Stage 4S NB is now defined by the International Neuroblastoma Staging System (INSS) as primitive tumor (stage 1 or 2) with dissemination restricted to the liver, skin, and bone marrow(less than10%),in children younger than 1 year of age at diagnosis. Bilateral synchronous, multifocal tumors have been included in the definition.[2] According to the International Neuroblastoma Risk Group(INRG) Staging System, the age is extended to 18 months for the stage 4S tumor which is now called stage MS. MIBG scintigraphy must be negative in bone and bone marrow.[3] Stage 4S NB is a metastatic disease associated with good survival, despite a large tumor burden, as spontaneous regression frequently occurs. It has the highest rate of spontaneous regression among malignant tumors. However, in some infants, particularly less than 2 months of age rapid disease progression can be observed with severe functional impairment, with an estimated mortality rate of 10% to 20%.Bilateral adrenal NB is rare.[1-3] The literature search revealed60 cases of bilateral adrenal neuroblastoma. We report a 1-day-old male with 4S bilateral adrenal neuroblastoma complicated by marked hepatomegaly that was managed by chemotherapy.

## CASE REPORT

A 1-day-old male neonate with birth weight of 2.5kg was admitted with marked abdominal distension noted at birth. The baby was active with stable general condition. Antenatal ultrasonography report revealed the diagnosis of neuroblastoma during the last trimester of pregnancy. On examination there was no jaundice or subcutaneous nodules. Upper abdomen was distended and revealed a firm, markedly enlarged liver with smooth surface and sharp margins, moving with respiration and reaching the lower abdomen. A distinct large abdominal mass in the left flank was also palpable. Complete blood counts, liver and renal function tests were normal. Serum vanillylmandelic acid (VMA) was 12 mg/24 hours. LDH-2692 U/L and serum Ferritin-400 ng/ml. Radiographs showed calcification in the left upper abdomen along with enlarged liver silhouette. Ultrasonography revealed hepatomegaly and bilaterally enlarged solid cystic masses in suprarenal areas, displacing the kidneys inferiorly. Contrast-enhanced computed tomography (CECT) scan of chest, abdomen and pelvis showed heterogeneous masses in both adrenal glands, larger on left side showing solid cystic areas and calcifications, measuring 60 mm x 48 mm. Right side lesion measured 31 mm x 23 mm (Fig. 1). Liver was diffusely enlarged with numerous hypodense lesions in both lobes suggestive of metastasis (Fig. 2). There was no retroperitoneal lymphadenopathy, pleural effusion, or bone marrow involvement.

**Figure:**
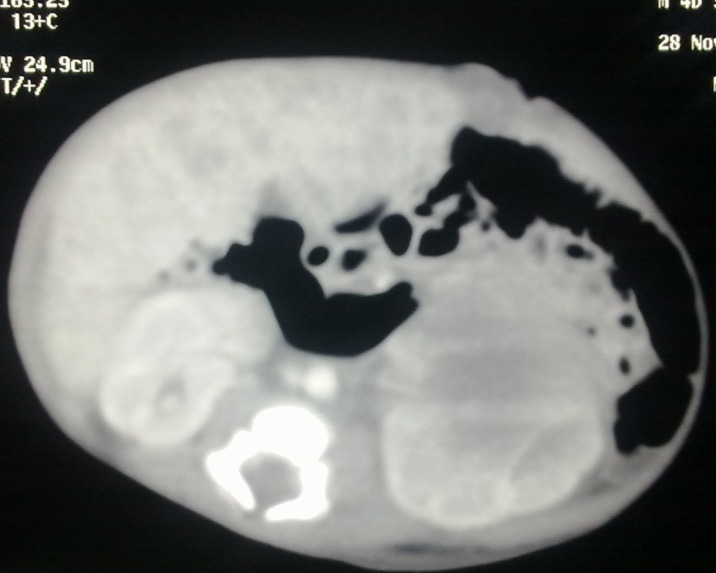
Figure 1:CT scan of abdomen showing heterogeneous masses in both suprarenal (adrenal glands), larger on left side showing solid cystic areas and calcifications, measuring 60 mm x 48 mm. Right side lesion measured 31 mm x 23 mm.

**Figure:**
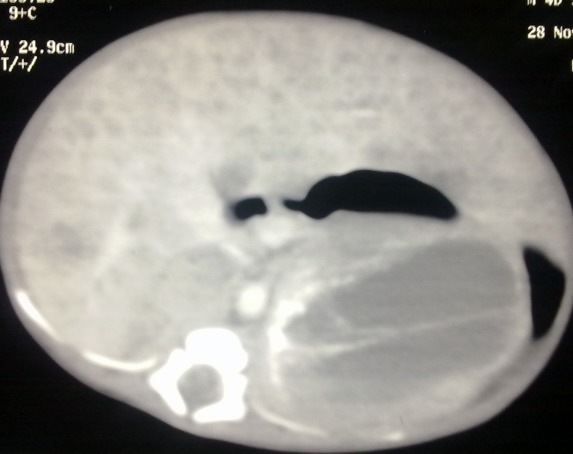
Figure 2:Liver was diffusely enlarged with numerous hypodense lesions in both lobes suggestive of metastasis.

Exploratory laparotomy was performed for surgical staging and tissue diagnosis. There was no ascites. A purple, enlarged liver with diffuse solid and cystic nodules on the surface were detected along with bilaterally enlarged adrenal glands. A biopsy was taken from the left adrenal for confirmation of the pathology. Hematoxylin and eosin (HE) staining revealed abundant clusters of small, round, blue cells with high nucleus-to-cytoplasm ratio and immature chromatin. Immunohistochemical (IHC) analysis was positive for chromogranin A (CgA) and synaptophysin. The final diagnosis was bilateral adrenal neuroblastoma, stage 4S with metastasis to the liver.

Patient was managed by adjuvant chemotherapy with 4 drugs, cyclophosphamide, cisplatin, etoposide, and doxorubicin over 8 months. There was complete regression of the liver metastasis along with calcification of the bilateral adrenal tumors. Three-year follow-up indicated neither recurrence nor residual tumors. Cardiomyopathy due to doxorubicin toxicity developed over ensuing years, which is being treated with digoxin, enalpril and frusemide. Child is otherwise active and playful.

## DISCUSSION

Treatment of 4S NB, particularly those younger than 2 months, with massive hepatomegaly, organ compromise and risk of death, is low-dose to moderate-dose chemotherapy with or without liver radiotherapy.[6] These patients benefit from prompt initiation of therapy, to push the NB cells towards the regression pathway. If this is achieved early, further intensive treatment may possibly be avoided.[1] It is difficult to identify infants with stage 4S disease, who will benefit from chemotherapy.[4] Those with favorable prognostic parameters should be kept under strict observation with supportive care for spontaneous regression to occur.[6]Resection of primary tumor is not associated with improved outcome.[6] A 4S NB complicated by massive hepatomegaly can be managed by abdominal decompression surgery. We operated child for diagnostic purpose as skin lesions and bone marrow involvement were not evident. Low dose radiotherapy to the liver is administered in doses of 100-150 Gy/day. Resolution of liver metastasis is probably related more to the natural course of stage 4S than to radiotherapy.[7] Most common cause of death in stage 4S neuroblastoma is hepatomegaly. Other causes are infection, disseminated intravascular coagulation, and radiation nephritis. Children with 4S neuroblastoma with bilateral adrenal tumors have a good prognosis.

To summarize, bilateral adrenal 4S NB may be noted on antenatal ultrasound. High index of suspicion is required in cases where isolated hepatic involvement is found. Biopsy may be required to make a definitive tissue diagnosis. Treatment with chemotherapy is sufficient to control such tumors.

## Footnotes

**Source of Support:** Nil

**Conflict of Interest:** None declared

